# Receptor Binding Domain-Specific B Cell Memory Responses Among Individuals Vaccinated Against SARS-CoV-2

**DOI:** 10.3390/vaccines12121396

**Published:** 2024-12-12

**Authors:** Atharv Athavale, Anmol Gaur, Nafees Ahmed, Adarsh Subramaniam, Jyotsna Dandotiya, Sneha Raj, Santosh Kumar Upadhyay, Sweety Samal, Anil Kumar Pandey, Ramesh Chandra Rai, Amit Awasthi

**Affiliations:** 1 BRIC-Translational Health Science and Technology Institute, Faridabad 21001, India; atharv1athavale@gmail.com (A.A.); anmolgaur454@gmail.com (A.G.); nafeesahmed23197@gmail.com (N.A.); adarsh.n@thsti.res.in (A.S.); jyotsna@thsti.res.in (J.D.); sneharaj@thsti.res.in (S.R.); sweety.samal@thsti.res.in (S.S.); 2 Department of Biotechnology, Kumaun University, Nainital 263001, India; upadhyaysk97@gmail.com; 3 ESIC Medical College and Hospital, Faridabad 121012, India; drpandeyak@yahoo.co.in

**Keywords:** receptor-binding domain (RBD), memory B cell (MBC), antibody-secreting cell (ASC), severe acute respiratory syndrome coronavirus 2 (SARS-CoV-2), coronavirus disease 2019 (COVID-19)

## Abstract

**Background**: The COVID-19 pandemic prompted unprecedented vaccine development efforts against SARS-CoV-2. India, which was one of the countries most impacted by COVID-19, developed its indigenous vaccine in addition to utilizing the ones developed by other countries. While antibody levels and neutralizing antibody titres are considered initial correlates of immune protection, long-term protection from the pathogen relies on memory B and T cells and their recall responses. In this regard, global research has primarily focused on mRNA-based vaccines. The studies on immune memory response, particularly B cell memory response induced by the vaccines given to Indians, remain relatively obscure. **Methods**: We assessed Receptor Binding Domain-specific memory B cells in the peripheral circulation and their ability to secrete antigen-specific antibodies among Indians vaccinated with Covaxin (BBV152), Covishield (AZD1222), Corbevax (BECOV2D), and Sputnik Light, as well as unvaccinated individuals. **Results**: Corbevax and Sputnik Light conferred better antibody-secreting cell (ASC) responses over time compared to other groups. **Conclusions**: These findings contribute to our understanding of vaccine-induced immune memory in the Indian population; providing insights that could inform future vaccine strategies.

## 1. Introduction

Vaccines are used to protect people from various diseases and to limit their transmission to a larger population. Since the inception of vaccination in 1796, there have been tremendous advancements in the field of vaccinology. From early whole-pathogen-inactivated vaccines to modern mRNA vaccines, the field has shown great progress, reaching a crucial turning point during the recent COVID-19 pandemic [1]. While vaccines against SARS-CoV-2 have proven their effectiveness in mitigating severe symptoms and reducing hospitalization, the cellular memory responses they induce remain largely uncharacterized [2,3]. Antibody responses, on the other hand, have been studied extensively. Notably, in addition to IgG, both IgA and IgM are capable of neutralizing viral antigens [4,5]. Besides antibodies, the generation and maintenance of immune memory is an important component of long-term protection, which involves T and B memory cells [6,7,8,9]. Cytotoxic CD8+ T cells eliminate the cells infected with an intracellular pathogen such as a virus, while CD4+ T cells generate a cytokine milieu conducive for B and T cell maturation and memory development [10,11]. Memory B cells (MBCs) are generated in response to an infection or vaccination and add a further layer of protection [12]. B cell affinity maturation occurs in the transient but specialized structures known as ‘germinal centres’ [13]. After a proliferative stage where a B cell clone undergoes somatic hypermutation and antigen-based selection, only those producing high-affinity antibodies survive and differentiate into MBCs [14,15]. This process continues for several months after vaccination or an infection, resulting in antibodies with improved avidity and neutralization capacity [8,15,16]. The affinity maturation process expands the breadth of recognition with increased affinity of the antibodies, and the generated pool of MBCs is multi-pronged with anticipatory memory potential [15].

A comparative assessment of memory B cell response elicited by different vaccines holds the potential to provide invaluable insights into their relative efficacies in promoting enduring immune memory, thereby facilitating the development of evidence-based vaccination strategies [17]. It is also important to assess the level of memory cells present in the peripheral circulation of vaccinees over time as well as their ability to respond to recurrent invasion by the pathogen [18]. There are concerns regarding the potential decline in vaccine efficacy, as viruses carrying mutations in key neutralizing antibody epitopes propagate in the community, allowing them to partially or completely evade antibody recognition [19,20]. There are also reports in certain cases of severe COVID-19, that the affinity maturation of B cells is compromised [21]. In India, several COVID-19 vaccines have been administered, including viral vector-based vaccines (Covishield, Sputnik Light, Sputnik V), an inactivated virus-based vaccine (Covaxin), and a protein subunit vaccine (Corbevax) [22,23,24,25,26]. After the promising results shown by these vaccines, with efficacies ranging from 70.4% to over 90%, their long-term effectiveness and the ability to generate immune memory are the areas of active research [27,28,29,30]. Despite the initial success, waning vaccine protection and breakthrough infections have been observed [31,32,33,34]. This is partly linked to SARS-CoV-2’s high mutation rate and immune evasion capabilities [35]. However, comprehensive data are not available to convincingly explain this immunological insufficiency that leads to pathogen’s upsurge with time, particularly in the Indian context.

The goal of this study was to address this knowledge gap by examining the diverse aspects of the antigen-specific B cell immune response three months after vaccination. Our readouts were based on the receptor-binding domain (RBD) of the spike protein of the SARS-CoV-2 Wuhan strain. It is well-established that the immune response against RBD has been shown to be dominant and correlates well with virus neutralization [18,36,37,38]. Our objective was to compare the memory B cell response generated by four vaccines—Covaxin (BBV152), Covishield (AZD1222), Corbevax (BECOV2D), and Sputnik Light—and the ability of these cells to proliferate and secrete anti-RBD antibodies. We characterized circulating memory B cells using the conventional combination of CD27 and IgD as phenotypic markers [39,40]. By comparing these diverse vaccines, which differ in their development platforms or dose regimens, we aimed to provide insights into their ability to confer enduring immunity.

## 2. Materials and Methods

### 2.1. Ethics Statement

This study was conducted at the BRIC-Translational Health Science and Technology Institute, Faridabad, India. Participant enrolment and sample collection were done at ESIC Medical College and Hospital, Faridabad. Written informed consent was obtained from each of the study participants. This study was approved by the institutional ethics committees of both institutes (ESIC Hospital and Medical College, Faridabad File no. 134 X/11/13/2021- IEC/43 and BRIC-THSTI Faridabad Ref No: THS 1.8.1/(130) dated 27 October 2021).

### 2.2. Participant Details

Samples were collected from a total of 171 participants, and their age, sample collection date, and vaccination details were recorded. The participants were of the following categories: unvaccinated (35 participants) and vaccinated (Corbevax—34, Covaxin—33, Covishield—40, and Sputnik Light—29) (Table 1). The sample collection for this study coincided largely with the Omicron-I (December 2021–June 2022) and partially with the Omicron-II (July 2022–October 2023) periods.

### 2.3. Inclusion and Exclusion Criteria

The analysis included participants with ages 18–59 years having no documented history of SARS-CoV-2 infection. Eligible individuals were either unvaccinated or had completed a full vaccination course (two doses for BBV152, AZD1222, and BECOV2D; a single dose for Sputnik Light). Participants were excluded if they had obtained a booster vaccine dose prior to specimen collection or had reported a prior infection at the time of enrolment. Pregnant women and people with immunocompromising comorbidities were also excluded from the study.

### 2.4. Blood Collection and Sample Processing

8 mL of blood was collected from each participant, in Greiner Bio-One tubes (Noida, India, Cat. No. 22-040-134) coated with sodium heparin. Blood samples were processed immediately to isolate plasma and peripheral blood mononuclear cells (PBMCs) using Lymphoprep density gradient centrifugation. Plasma was stored at −80 °C, and PBMCs were resuspended in fetal bovine serum (FBS) supplemented with 10% dimethyl sulfoxide (DMSO) and then stored in liquid nitrogen [16].

### 2.5. Indirect Anti-RBD IgG ELISA

96-well Maxisorp plates (Thermo Fisher Scientific, Roskilde, Denmark-Cat No. 442404) were coated with 100 µL of RBD protein (2 µg/mL in PBS) per well and incubated overnight at 4 °C. Plates were washed with PBST (PBS + 0.1% Tween 20) and blocked for two hours at 37 °C with a block buffer (PBST + 3% skimmed milk). After incubation, the block buffer was discarded, and 100 µL per well of the diluted plasma samples were added in duplicates. Plasma samples were diluted in PBST + 3% skimmed milk at a ratio of 1:150. The plate was incubated for 30 min at 37 °C and then was washed with PBST. Further, 100 µL of secondary antibodies was added per well [Goat anti-human IgA HRP-conjugated (Southern Biotech, Birmingham, AL, USA, 2050-05; 1:5000 dilution), goat anti-human IgG HRP-conjugated (Jackson Immunoresearch, West Grove, PA, USA, 109-035-088; 1:10,000 dilution), and goat anti-human IgM HRP-conjugated (Jackson Immunoresearch 109-035-129; 1:10,000 dilution)]. The plate was incubated for 30 min at room temperature and then washed with PBST. TMB substrate was added in the dark and was incubated for 3 min. The reaction was stopped with 1 N H_2_SO_4_ before measuring the optical densities at 450 nm using a microplate reader [41].

### 2.6. Expression and Purification of the RBD Protein of SARS-CoV-2 (Wuhan Strain-Hu-1)

The following reagent was contributed by David Veesler for distribution through BEI Resources, NIAID, NIH: Vector pcDNA3.1(-) containing the SARS-related coronavirus 2, Wuhan-Hu-1 spike glycoprotein receptor-binding domain (RBD), and NR-52422as, as mentioned previously [42,43]. The recombinant his-tagged SARS-CoV-2 ancestral Wu-RBD protein was expressed in transiently transfected Expi293F cells in suspension culture using an Expifectamine transfection kit (Thermo Fisher Scientific, Cat no. A14524) as per the manufacturer’s protocol. Post-transfection and expression, cell culture supernatants were harvested after 5–6 days or until cells showed more than 60% cell death. The supernatant was passed through a Ni–NTA column for protein purification. Bound proteins were eluted with 500 mM imidazole and were concentrated with an Amicon 10 kDa filter (Millipore, Burlington, MA, USA). Protein fractions were aliquoted and stored at −80 °C.

### 2.7. Labelling of RBD Protein with the Alexa Fluor-488

Purified RBD was tested using SDS PAGE to verify its purity and was used for the fluorophore labelling at a concentration of 1 mg/mL. Labelling of the RBD protein was performed using the Alexa Fluor Microscale Protein Labelling kit (Cat No. #A30006; Thermo Fisher Scientific) as per the manufacturer’s instructions. Fluorophore-labelled RBDs were aliquoted and stored at −20 °C for further use.

### 2.8. Estimation of Antibody-Secreting Cells

Antigen-specific antibody-secreting cell estimation was performed as described by Crotty et al., 2004 [16] with slight modifications. PBMCs were washed after thawing with a complete RPMI medium, counted and cultured at a density of 3 million cells per sample, and added at 0.5 million cells per well in a 24-well plate. An additional 0.5 million cells were cultured in a medium without stimulants as a control. The polyclonal stimulation reagents used were protein A from *Staphylococcus aureus* (Sigma Aldrich, St. Louis, MO, USA, P7155), lectin from *Phytolacca americana* (pokeweed) (L9379), and ODN 2006 (TLR GRADE^®^) (synthetic) (Enzo Life Sciences, Farmingdale, NY, USA, -ALX-746-056-M001). The culture plate was kept in a CO_2_ incubator for 5 days at 37 °C.

An ELISpot plate (Sigma Aldrich-MSIPS4510) was activated with 35% ethanol. After washing with PBST (PBS + 0.05% Tween 20) and PBS, the plate was coated with polyvalent goat anti-human Ig mix (Thermo Fisher Scientific—H17000) and SARS-CoV-2 spike RBD at a concentration of 10 μg/mL in PBS. The plate was incubated overnight in the dark at 4 °C. Following coating, the plate was washed and blocked with 200 µL of a blocking buffer per well for at least 2 h at 37 °C or overnight at 4 °C. After culturing for 5 days, cells were washed and seeded onto the ELISpot plate (Section 3.3). Subsequent steps included incubation with secondary antibodies [(goat anti-human IgA secondary antibody, Biotin (A18785), goat anti-human IgG Fc Bio Affinity (Thermo Fisher Scientific—A18821), goat anti-human IgM secondary antibody, Biotin (Thermo Fisher Scientific—PA1-86071)], Avidin-D-HRP (Thermo Fisher Scientific 18-4100-51), and a substrate solution. For every plate, the substrate was prepared by mixing 110 µL of an AEC-DMF solution (3-Amino-9-Ethylcarbazole in Dimethyl Formamide, 60 mg/mL) with 10 mL of 0.1 M sodium acetate and adding 165 µL of 3% H_2_O_2_ (*w*/*v*) [Sigma Aldrich (323381)] after filtering the solution to remove any particulates. The plate was incubated for development of spots, rinsed gently with tap water, and dried overnight in the dark.

Spot counting was performed using the auto-counter feature of the CTL ImmunoSpot 7.0.36.0 device, which visualized and recorded the number of spots in each well. Quality control was performed to check for any errors in the counting, and corrections were made accordingly. The average of the spot-forming units (SFUs) from the unstimulated control wells was deducted from each of the stimulated sample SFU values.

### 2.9. Estimation of Memory B Cells in the Peripheral Circulation

To estimate the percentage of RBD-specific memory B cells in the peripheral circulation of the study participants, a flow-cytometry-based method was utilized [18]. Flow cytometry was performed on a subset of samples only when sufficient PBMCs remained after culturing them for the ELISPOT assay. Alexa Fluor-488 (Invitrogen, Carlsbad, CA, USA, #A30006) fluorophore-labelled RBD was used as a probe for estimation of antigen-specific memory B cells. Briefly, 2 × 10^6^ cells were first labelled with fixable viability dye (Invitrogen #L34964) and incubated for 15 min at 4 °C to distinguish between live and dead cells. The cells were washed twice by centrifugation with FACS buffer (1% FBS in PBS) and subsequently stained with antibodies targeting molecular markers that can differentiate memory B cells among PBMCs (Table 2). After incubation at 4 °C for 30 min and washing with FACS buffer, samples were acquired using a FACSCanto II flow cytometer (BD Biosciences, San Jose, CA, USA). The data were analyzed using FlowJo 10.3.0 (FlowJo LLC, Ashland, OR, USA). The gating strategy used was CD3^−^ (exclusion of T cells), CD19^+^ and CD20^+^ (pan-B cell markers), CD27^+^IgD^−^ (class-switched memory B cells), and RBD^+^ (antigen-specific memory B cells).

### 2.10. Statistics

All the data visualization and statistical analyses were performed using GraphPad Prism 10.2.0. Data were checked for normal distribution before analysis. The outliers were removed using the iterative Grubb’s test (Alpha = 0.01). Datasets were visualized as bar charts depicting the median and interquartile range. Depending on the data, Mann–Whitney U/Wilcoxon rank-sum, Kruskal–Wallis, and Dunn’s multiple comparison tests were used for statistical analyses, as appropriate. A *p* value of <0.05 was considered statistically significant.

## 3. Results

### 3.1. All Participants, Including Unvaccinated Individuals, Showed Marked Levels of Anti-RBD IgG Antibodies

**Figure 1:**
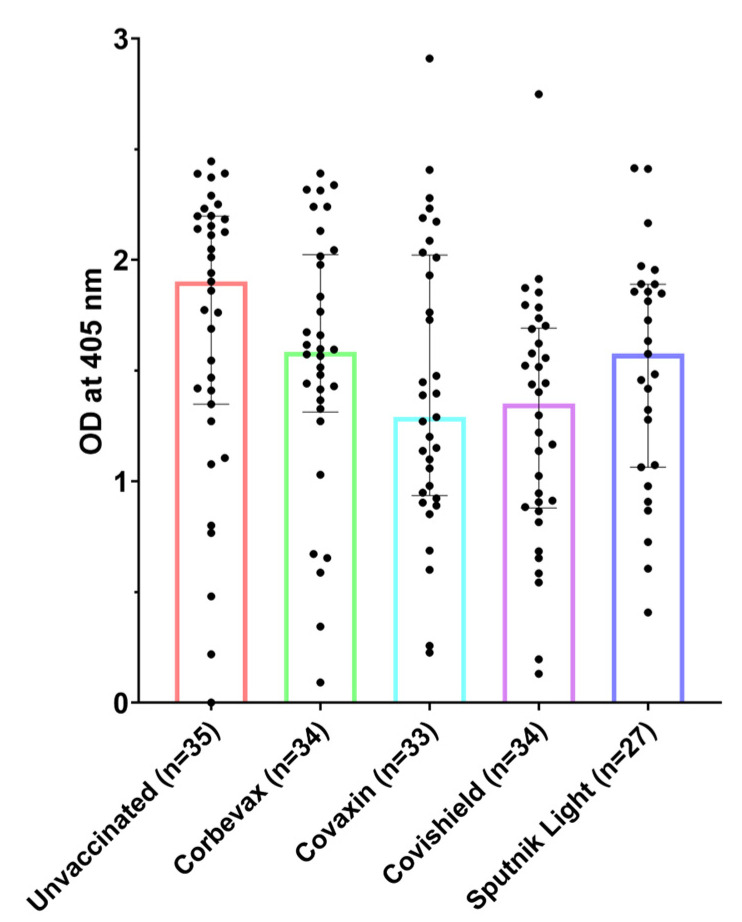
IgG antibody levels against SARS-CoV-2 receptor-binding domain (RBD) of spike protein among unvaccinated and vaccinated participants.

The IgG antibody levels of the participants in our study were tested using RBD-specific IgG ELISA [41]. Vaccinated participants from all four vaccine groups showed a remarkable IgG response towards SARS-CoV-2 RBD, indicating that the vaccinations led to the development of a distinct humoral response against the virus (Figure 1). However, the unvaccinated participants also showed an elevated RBD-specific IgG response, which suggests their possible exposure to SARS-CoV-2 during the pandemic. For this study, the blood samples from unvaccinated participants were collected during the Omicron surge in India, so it is likely that these participants contracted the virus during this period and remained asymptomatic [44]. The presence of post-recovery IgM antibodies for a prolonged time among these participants highlights the importance of these multimeric antibodies in tackling the virus. Similar observations have been made in other viral infections, where the higher avidity of these antibodies towards the antigens enables hosts to continue producing them in the backdrop of the persistent antigenic challenge [4,45].

### 3.2. Study Participants Exhibited RBD-Specific Memory B Cells in the Peripheral Circulation

**Figure 2:**
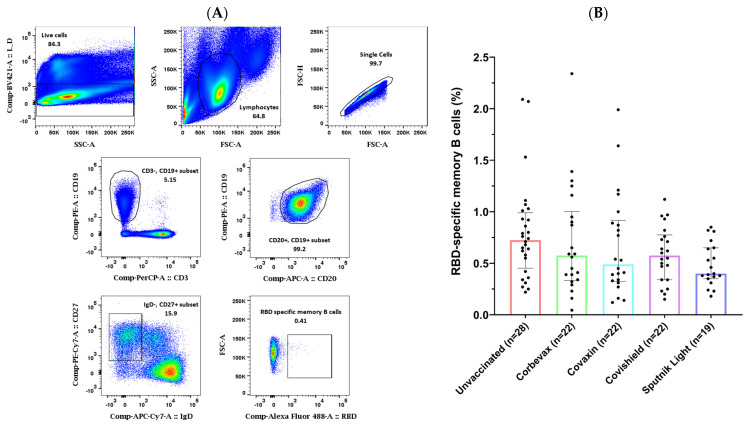
(**A**) Representative flow cytometry gating strategy for SARS-CoV-2 RBD-specific memory B cells (CD3^−^, CD19^+^, CD20^+^, IgD^−^, CD27^+^, RBD^+^). (**B**) Graphical representation comparing the percentage of RBD-specific memory B cells in vaccinated and unvaccinated participants [outliers removed using iterative Grubb’s test, alpha = 0.01].

Our analysis of the MBC responses to the SARS-CoV-2 RBD in the peripheral blood of the participants yielded mixed results. Flow cytometry analysis on PBMCs from 113 participants (85 vaccinated and 28 unvaccinated) revealed a higher percentage of RBD-specific memory B cells in the unvaccinated group as compared to any of the vaccinated groups. Although we recruited these unvaccinated participants based on their asymptomatic profile during the last three months, they showed a good antibody response against SARS-CoV-2 RBD. This is probably because of a fresh natural (asymptomatic) exposure of the unvaccinated participants to the pathogen at the time of the Omicron surge in the country [44] (Figure 2). All the participants of vaccinated groups showed comparable percentages of RBD-specific memory B cells in the peripheral blood. Similar to our observation for the RBD-specific antibody levels in the plasma samples, the group of unvaccinated participants had a slightly higher number of RBD-specific memory B cells as compared to all the vaccinated groups in the peripheral circulation. However, there was no statistically significant difference seen among participants of vaccinated groups when compared with that of the unvaccinated participants.

### 3.3. Magnitude of the RBD-Specific Antibody-Secreting B Cells Among Vaccinees from Different Vaccine Groups

**Figure 3:**
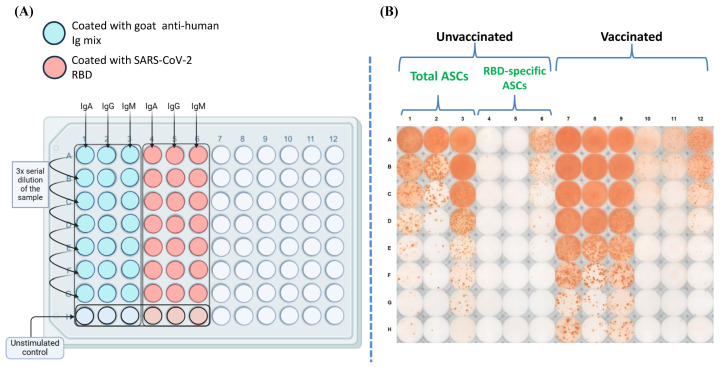
(**A**) Schematic ELISPOT assay plate map (created using BioRender). (**B**) Representative plate image showing the total and RBD-specific antibody-secreting cells (ASCs) among the cells collected from an unvaccinated and a vaccinated individual.

**Figure 4:**
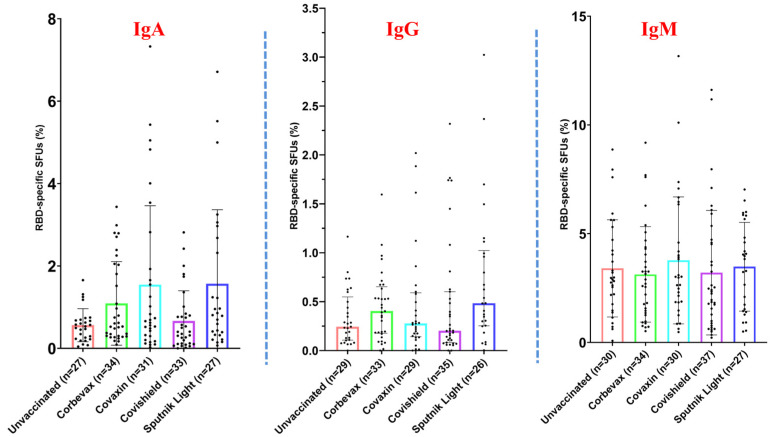
Fraction of the RBD-specific ASCs (IgA, IgG, and IgM) among vaccinated versus unvaccinated individuals [outliers removed using iterative Grubb’s test, alpha = 0.01].

To evaluate the magnitude of the RBD-specific antibody-secreting B cells among vaccinees from different vaccine groups, polyclonal stimulation of the PBMCs was performed, and the ability of the B cells to proliferate and secrete antigen-specific antibodies was tested [16]. The schematic diagram of the plate map and a representative image of the B cell ELISPOT plate are shown in Figure 3A and Figure 3B respectively. Our data revealed a distinct distribution of RBD-specific IgM, IgA, and IgG antibody-secreting B cells among the participants. The percentage of RBD-specific IgM antibody-secreting B cells was higher than that of IgA and IgG antibody-secreting B cells across all the analyzed groups (Figure 4). However, it was almost similar across all the participant groups. The median percentage of IgA and IgG antibody-secreting B cells was slightly higher among participants vaccinated with the Corbevax and Sputnik Light as compared with the other groups, including the unvaccinated participants. Covaxin also mounted a slightly better IgA response compared to the unvaccinated group. There were no statistically significant differences in the ASC response between the groups because of considerable variability, as evident from Figure 4. The increase in the median percentage of IgA and IgG antibody-secreting B cells in the participants of the Corbevax and Sputnik Light groups, as well as the IgA response in the Covaxin-vaccinated participants, is of significance when considering the vaccine-induced immune memory response. Even though these groups had a lower level of RBD-specific IgG antibodies in their plasma samples (Figure 1) and fewer RBD-specific memory B cells in circulation compared to the unvaccinated group (Figure 2), a reversal in the pattern was observed in their ASC responses. It should be noted that a single-dose Sputnik Light vaccine, resulted in a marked level of the RBD-specific IgG antibody-secreting B cells comparable to those produced by other vaccines after two doses.

### 3.4. Temporal Patterns in the RBD-Specific Antibody-Secreting B Cells Post-Vaccination

**Figure 5:**
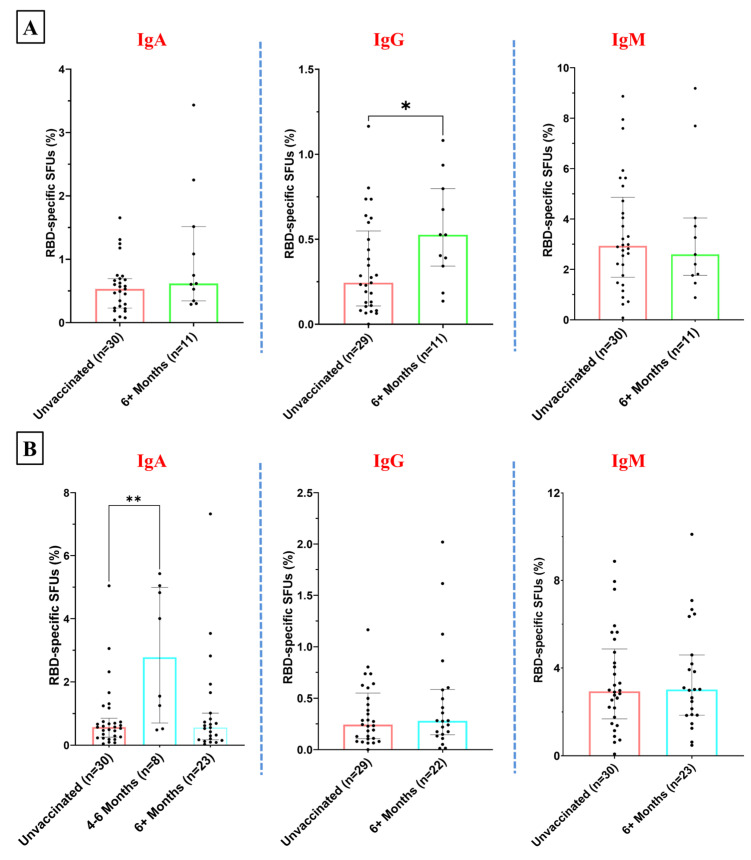

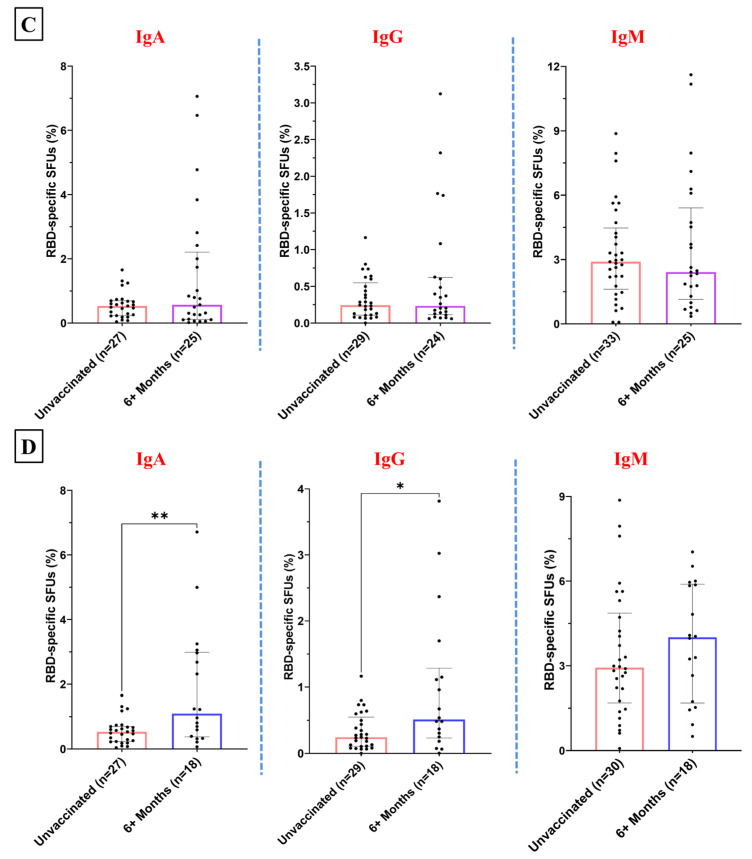
Fraction of the RBD-specific ASCs (IgA, IgG, and IgM) to depict temporal changes (for participants vaccinated more than 6 months prior to sample collection): (**A**) among Corbevax-vaccinated versus unvaccinated individuals, (**B**) among Covaxin-vaccinated versus unvaccinated individuals, (**C**) among Covishield-vaccinated versus unvaccinated individuals, and (**D**) among Sputnik-Light-vaccinated versus unvaccinated individuals [Mann–Whitney U test; * *p* < 0.05, ** *p* < 0.01; outliers were removed using iterative Grubb’s test, alpha = 0.01].

To test the durability of the ASC response of the participants of different vaccine groups, we categorized the samples of each vaccination group by 4–6 months and beyond 6 months post-vaccination. The levels of IgA- and IgM-secreting B cells among the Corbevax-immunized participants post-6-months of vaccination did not differ significantly from the participants in the unvaccinated group (Figure 5A). However, they exhibited a statistically significant increase in RBD-specific IgG ASC suggesting an enhanced IgG antibody response among these participants over time (Figure 5A). However, at early time points, a slight enhancement in the median of the ASC response for only IgG was observed among the Corbevax-vaccinated participants (Supplementary Figure S1). The ASC response after Covaxin administration remained similar to that of the unvaccinated group among the samples obtained post-6-months of vaccination (Figure 5B). We observed an increase in the median RBD-specific IgA and IgG SFUs at an early time point with a significant inter-individual variation (Supplementary Figure S2). However, the above result cannot be considered conclusive, as the number of samples in the 4–6 months post-vaccination group is not sufficient. Participants immunized with Covishield did not show a noticeable trend in the memory B cell immune response. The percentage of RBD-specific antibody-secreting B cells remained similar for 4–6 months and thereafter in these participants (Figure 5C and Supplementary Figure S3). Individuals who received the Sputnik Light vaccine displayed an enhancement in the levels of RBD-specific IgA and IgG antibody-secreting B cells after 6 months post-vaccination (Figure 5D). However, we did not observe a marked difference in this regard at early time points (Supplementary Figure S4).

This observation suggests the development of a durable and broader antibody response over time following Corbevax and Sputnik Light vaccination. A limitation in drawing definitive conclusions about the impact of various vaccines on the temporal dynamics of ASC responses is the relatively small number of samples collected after vaccination with Covaxin, Covishield, and Sputnik Light within the 4- to 6-month time window (Supplementary Figures S1–S4).

### 3.5. No Sex-Specific Disparities Observed in Serological or Memory Immune Responses

**Figure 6:**
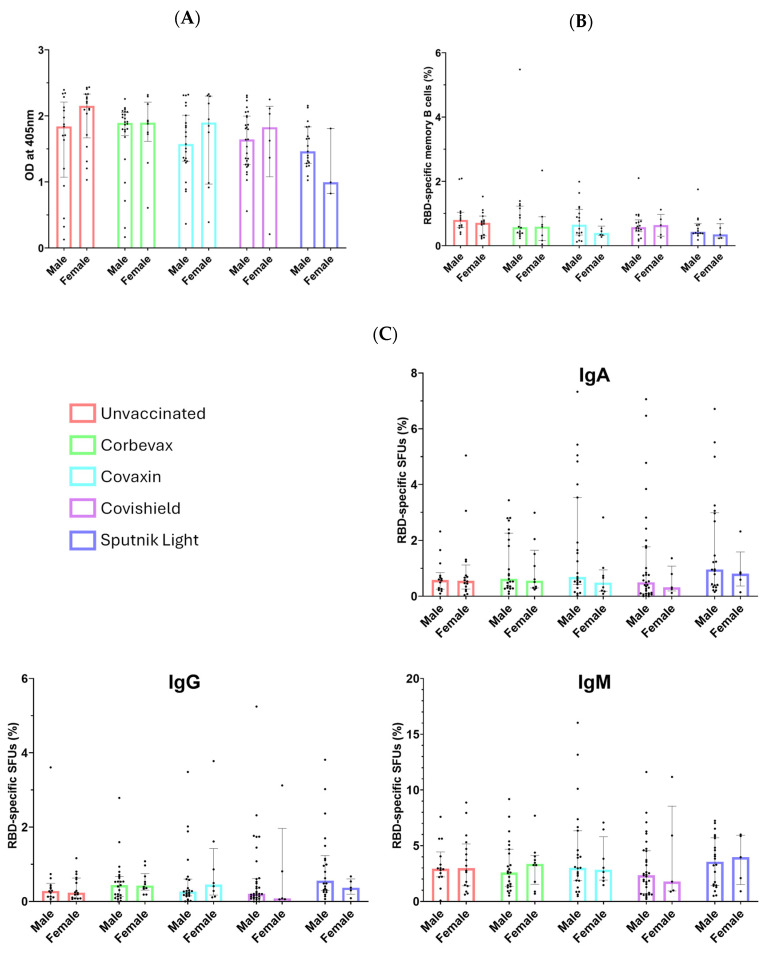
Gender-disaggregated analysis of immune response and memory B cell response. (**A**) IgG antibody levels against SARS-CoV-2 spike RBD compared between male and female participants. (**B**) Percentage of RBD-specific memory B cells in the peripheral circulation of male and female participants segregated based on the vaccine given. (**C**) Percentage of RBD-specific IgA, IgG, and IgM spot-forming units in unvaccinated and vaccinated males and females.

A considerable number of males and females participated in this study, as evident from Table 1B. We performed a sex-disaggregated analysis of the immune responses to evaluate any disparities between males and females. As shown in Figure 6, our analysis revealed no statistically significant differences in serological immune responses or B cell memory responses between male and female participants. This finding indicates that sex does not appear to influence the B cell immune response to vaccination. Despite the lower number of vaccinated females compared to males, our data suggest that the immune responses are comparable across sexes.

## 4. Discussion

Understanding the immune memory response is crucial for gaining insights into the longevity of the protection against pathogens. The memory responses of T and B cells and their functionality are shaped not only by the antigens of a pathogen and their presentations but also by the interactions between these cell types [46]. The B cell memory phenotype is generated as a result of multiple iterations of antigen-based selection of antibody-secreting B cell populations [47]. The instability of the viral genome and the resulting mutations in the antigens, particularly the spike protein in the case of SARS-CoV-2, diminishes the impact of B cell memory response during a reinfection or a breakthrough infection [48]. It has been shown that MBC responses were more pronounced when individuals experienced a natural infection followed by a single vaccine dose as opposed to vaccination alone [49]. In our study, it was observed that even the unvaccinated individuals exhibited antibody response as well as memory B cells with proliferative capability in their peripheral circulation, likely due to asymptomatic infections during the Omicron wave [44]. This highlights the complex interplay between vaccination, prior infection, virus-specific MBC generation, and maintenance.

The generation and durability of MBCs has been linked to the severity of the disease, where moderate to severe infections have resulted in generating the ability to mount a better B cell recall response. Several studies show that the overall antibody titres decline rapidly a few months following infection and vaccination after attaining a peak in the initial days [50,51,52]. Relatively few studies have been performed to gain insight into the protection provided by memory B cells. Most of the studies in this field have focused on the mRNA-based vaccines [53,54,55,56,57,58]. Various aspects of immunological memory produced by BNT162b2 (Pfizer-BioNTech) vaccination against SARS-CoV-2 have been widely studied [57,59,60]. In India, where the BNT162b2 vaccine was rarely administered, such studies are lacking.

Among the vaccinated groups, we found variations in the fraction of ASCs induced by different vaccines. Notably, BECOV2D and Gam-COVID-Vac showed promising results, with a slight increase in the number of ASCs six months after vaccination, similar to what was previously observed in BNT162b2 [59,61]. On the other hand, though BBV152 and AZD1222 induced a considerable number of ASCs after vaccination, they did not show any significant change in the B cell recall response over time. These vaccines induced a B cell recall response like a mild infection, but the temporal dynamics depended on the specific vaccine. Although some of the vaccinated individuals got infected during the subsequent waves of COVID-19, it is well established that vaccination reduced the severity of the disease and hospitalizations. This is possibly because of the broader cellular memory responses generated by these vaccines which tackled the virus in an efficient manner and hence reduced the disease severity [3].

Our study has some limitations that should be addressed in future research. First, a larger sample size would provide a clear understanding of the temporal dynamics of the ASC response mounted by different vaccines. It would have been ideal to have access to pre-pandemic PBMCs and plasma samples. The lack of such samples limits our ability to distinguish between infection-induced and infection as well as vaccination-induced responses. Additionally, our study solely focused on B cell responses. Although studies have analyzed T cell memory response by different vaccines, analysis of both T and B cell memory responses on the same group of participants would add a holistic understanding of the immune memory response mounted by vaccination. Further, we observed a substantial interindividual variation among these responses at the level of RBD-specific memory B cells in the peripheral circulation as well as in the secretion of antigen-specific antibodies, reflecting immunological heterogeneity among humans [62]. While the MBC phenotyping method we followed (CD3^−^, CD19^+^, CD20^+^, CD27^+^, IgD^−^) is widely accepted, recent research suggests that CD27 may not be able to serve as an ideal marker for circulating MBCs [63,64]. Despite these limitations, this study improves our understanding of the memory B cell response induced by various COVID-19 vaccines used in India, an aspect which was previously underexplored [65,66].

## 5. Conclusions

The levels of memory B cells generated by vaccination or natural infection serve as a better immune correlate of protection than antibody levels in the blood circulation [67]. Our study highlights the immune memory response, particularly the B cell memory response, generated after vaccination against COVID-19. Although we did not find any significant difference between the MBC responses of vaccinated and unvaccinated (presumably infected) participants, it should be noted that vaccinated individuals demonstrated an enhanced ability to secrete RBD-specific antibodies during re-exposure. This suggests that while vaccination may not significantly alter MBC numbers in a highly exposed population, it does improve the breadth and magnitude of the B cell recall response. A high percentage of individuals infected during the Omicron wave in India remained asymptomatic [44]. This is potentially the reason for the unvaccinated individuals exhibiting high levels of RBD-specific IgG antibodies, as observed in this study. They also showed the presence of RBD-specific B cells in their peripheral circulation. However, the vaccinated participants, especially those vaccinated with Corbevax and Sputnik Light, showed better response in terms of secreting RBD-specific antibodies after polyclonal stimulation. This suggests an effective and durable B cell memory response generated by these vaccines. These findings contribute to our understanding of vaccine-induced immune memory and may inform future vaccination strategies, including the potential need for booster doses. This knowledge could also help government bodies in making policy decisions, including the preferential use of a particular vaccine development platform. This study is also unique in guiding tailored and more equitable vaccination strategies based on the understanding of the durability of the memory response of B cells and their ability to proliferate and secrete antigen-specific antibodies. Further research is needed to fully evaluate the long-term protection conferred by these vaccines, especially in the context of emerging SARS-CoV-2 variants. Our study underscores the importance of continued research into vaccine-induced immune responses to guide public health strategies in the management of COVID-19 or similar infectious diseases in the future.

## Figures and Tables

**Table 1:** Participant details (A) and demography (B).

(A)						
Sr. No.	Status of Vaccination				Number	
1	Unvaccinated				35	
2	Vaccinated (Corbevax)				34	
3	Vaccinated (Covaxin)				33	
4	Vaccinated (Covishield)				40	
5	Vaccinated (Sputnik Light)				29	
**(B)**						
		**Unvaccinated**	**Corbevax**	**Covishield**	**Covaxin**	**Sputnik Light**
Sex	Male	17	24	33	24	25
	Female	18	10	7	9	4
Age	Range	37 (18–55)	26 (20–46)	34 (19–53)	33 (21–54)	39 (20–59)
	Median	28	29	26	26	30
Months since vaccination	Range	-	5 (3–8)	12 (3–15)	12 (3–15)	5 (6–11)
	Median	-	6	8	11	8

**Table 2:** Molecular markers used for the memory B cell estimation in the peripheral circulation of the study participants.

SN.	Molecular Marker	Fluorophore	Make and Catalogue Number
1	Fixable viability dye	Violet Dye	Invitrogen, Waltham, MA, USA #L34964
2	CD3	PerCP	BioLegend, San Diego, CA, USA #344814
3	CD19	PE	BioLegend, San Diego, CA, USA #302208
4	CD20	APC	BioLegend, San Diego, CA, USA #302310
5	CD27	PE-Cy7	BioLegend, San Diego, CA, USA #356412
6	IgD	APC-Cy7	BioLegend, San Diego, CA, USA #348218

## Data Availability

The authors will provide data while keeping the participants’ details confidential and as per the journal policy.

## Supplementary Materials

The following supporting information can be downloaded at: https://www.mdpi.com/article/10.3390/vaccines12121396/s1, Figures S1–S4: Fraction of the RBD specific ASC (IgA, IgG and IgM) to depict temporal changes at 4–6 months. Figure S1: among Corbevax vaccinated versus unvaccinated individuals. Figure S2: among Covaxin vaccinated versus unvaccinated individuals. Figure S3: among Covishield vaccinated versus unvaccinated individuals. Figure S4: among Sputnik Light vaccinated versus unvaccinated individuals.
