# Supplementary material for: Receptor Binding Domain-Specific B Cell Memory Responses Among Individuals Vaccinated Against SARS-CoV-2
Source: Vaccines (Basel). 2024 Dec 12;12(12):1396. doi: 10.3390/vaccines12121396 (PMC11680197; doi:10.3390/vaccines12121396)
Supplement: Supplementary file 1 [file vaccines-12-01396-s001.zip › vaccines-3228789-supplementary.pdf]

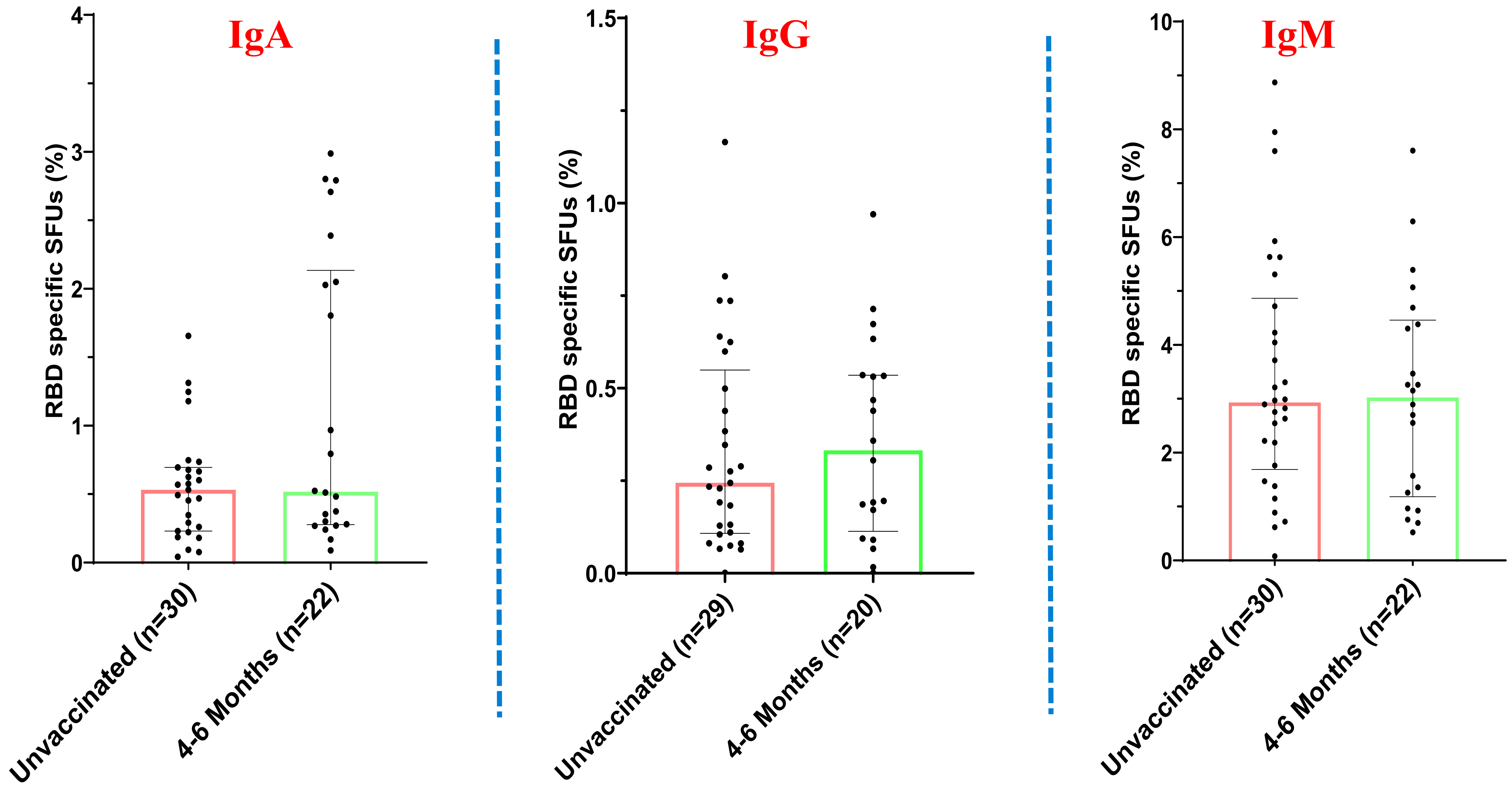

Supplementary Figure S1

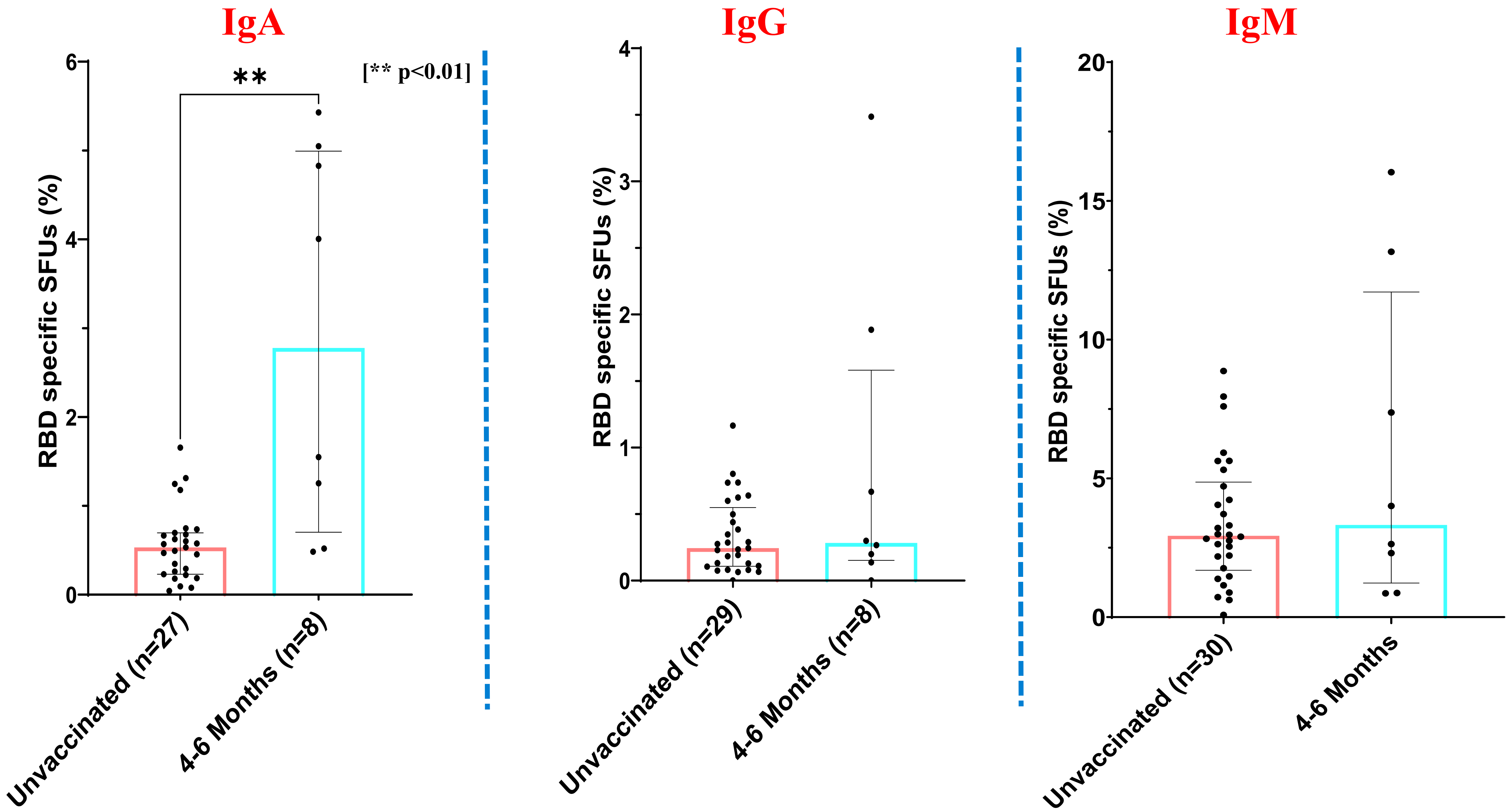

Supplementary Figure S2

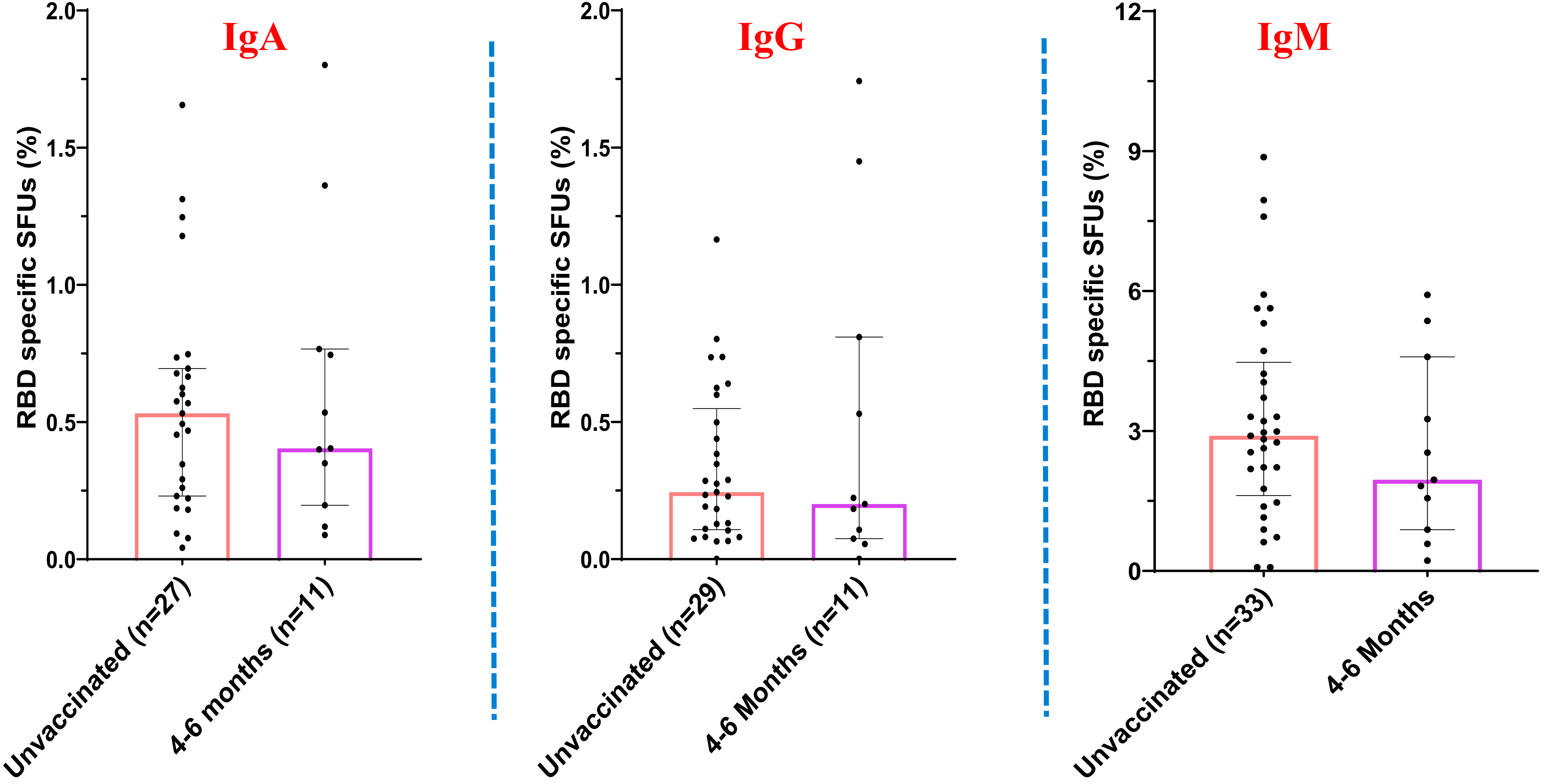

Supplementary Figure S3

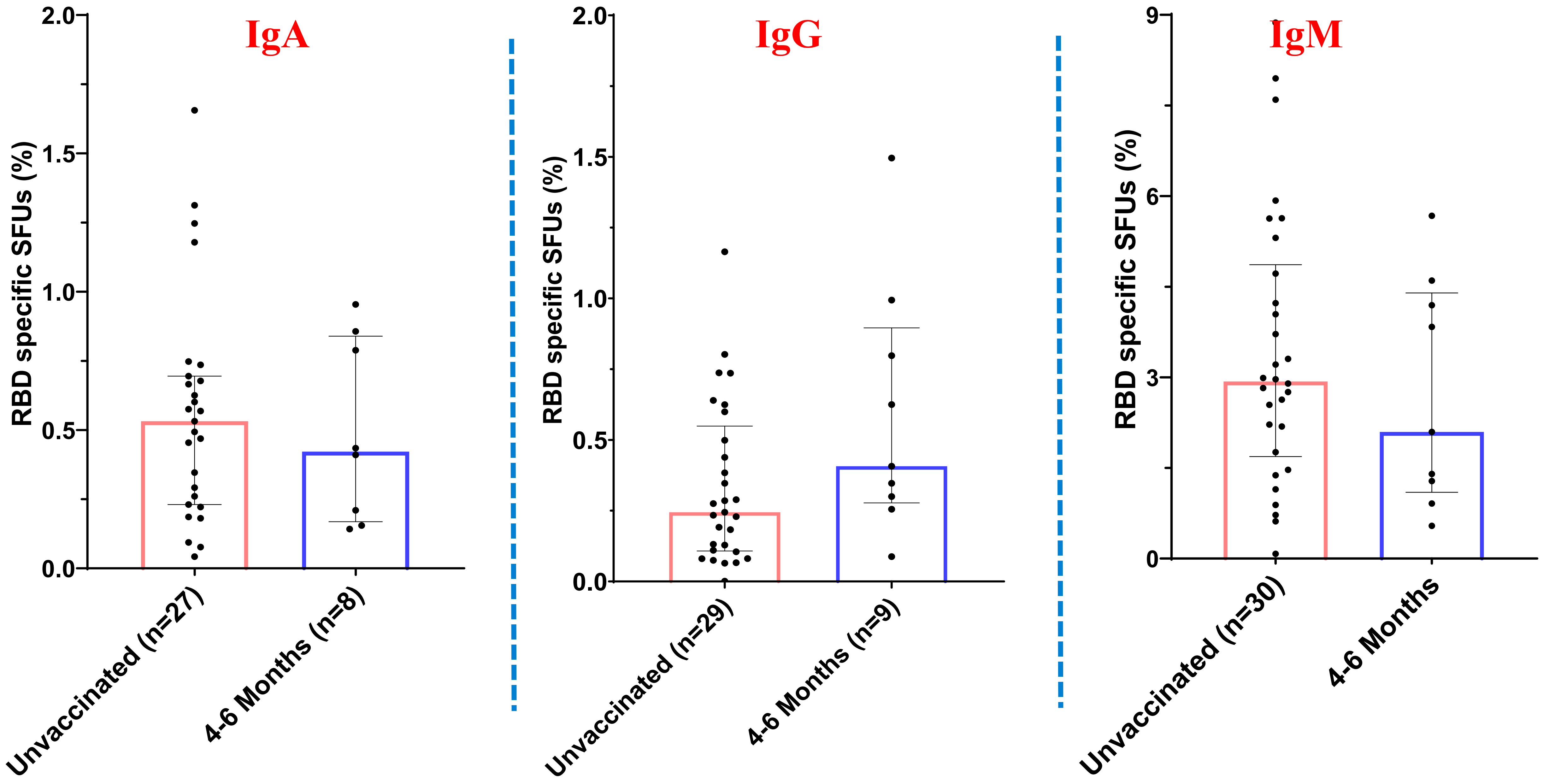

Supplementary Figure S4

Supplementary Figures S1-S4: Fraction of the RBD specific ASC (IgA, IgG and IgM) to depict temporal changes at 4-6 months. **Figures S1.** among Corbevax vaccinated versus unvaccinated individuals. **Figures S2.** among Covaxin vaccinated versus unvaccinated individuals. **Figures S3.** among Covishield vaccinated versus unvaccinated individuals. **Figures S4.** among Sputnik Light vaccinated versus unvaccinated individuals.
